# The status and associated factors of early childhood caries among 3- to 5-year-old children in Guangdong, Southern China: a provincial cross-sectional survey

**DOI:** 10.1186/s12903-020-01253-w

**Published:** 2020-09-25

**Authors:** Jianbo Li, Weihua Fan, Yueshan Zhou, Linmei Wu, Wei Liu, Shaohong Huang

**Affiliations:** 1 Stomatological Hospital, Southern Medical University, No. 366, South of Jiangnan Avenue, Guangzhou, China; 2 Faculty of School Health, Guangzhou Center for Disease Control and Prevention, Guangzhou, China

**Keywords:** Cross-sectional survey, Early childhood caries, Oral epidemiology, Preschool children, Associated factors

## Abstract

**Background:**

Dental caries of deciduous teeth (Early Childhood Caries, ECC) has become a crucial oral health problem over the decades in China. The aim of this study was to examine the prevalence and severity of ECC among preschool children from Guangdong Province, Southern China. In addition, to assess the association of ECC with reported oral health-related behaviors.

**Methods:**

A cross-sectional survey of 2592 participants was carried out in Guangdong Province by means of an equal-sized, stratified, multistage random sampling method during December 2015 and April 2016. The participants were divided into three groups according to their ages (3-, 4-, and 5-year-olds). Half of the participants were derived from urban areas, while the other from rural areas. According to the standard for clinical dentition examination of the WHO 2013 criteria, the presence of ECC was determined by the dmft (decayed-missing-filled tooth) index using a CPI (Community Periodontal Index) probe. A questionnaire about caries-related factors was completed by each of the participants’ parents or grandparents through a face-to-face and one-on-one interview. Then, *t*-test, Chi^2^ tests, One-Way ANOVA served for statistical analysis, and logistic regression analysis as well as covariance analysis were executed to identify potential associated factors for ECC.

**Results:**

The prevalence (% dmft > 0) of ECC was 68.3 (95% CI: 66.5–70.1), the mean dmft was 4.36 (95% CI: 4.17–4.55), and the filled rate was 1.2%. In multivariable modeling, associated factors for both prevalence and mean dmft were older age, rural areas, consumption of sweets before sleep, dental visit history, low household income, and low parental education level. Initiating toothbrushing after 3 years of age and being exclusively/ predominantly breastfed indicated only the prevalence; being female and frequently consuming sweetened milk/powdered milk indicated only the mean dmft.

**Conclusions:**

Preschool children in Guangdong Province, especially children from rural areas, experienced a significant amount of ECC. Associated factors for ECC included demographics, oral health measures, dietary factors, and socioeconomic factors. More attention should be given to prevention of ECC from early life. The construction of social support for oral health should be strengthened. Oral health education and promotion, especially of rural areas, should be intensified to reduce the inequality between urban and rural areas.

## Background

The term “early childhood caries” (ECC) is used to describe “any dental caries of deciduous teeth that occur in children ≤71 months, including one or more lesions (with or without formation of cavities), or leading to missing teeth, or leading to fillings on the tooth surface” [1]. ECC had impacts on eating patterns, permanent dentition development and the general health status of children [2]. ECC is a complex multifactorial disease and is impacted by numerous factors, such as dietary factors, oral health behaviors, socioeconomic factors and biological factors [3].

In the past 30 years, two provincial oral health surveys were executed in Guangdong. The survey in 1995 showed that the prevalence of ECC was 76.8% (mean dmft 4.81) among 5-year-old children, and the survey in 2005 showed that the prevalence was 67.8% (mean dmft 4.10) [4]. A declining but still strong trend was shown. ECC is a crucial oral public health problem in Guangdong. Moreover, ECC affects a considerable proportion of preschool children globally [5]. A goal of a 50% caries-free rate among 5-year-old children for the year 2020 was set by the WHO in 2003 [6]. Accordingly, appropriate techniques for the prevention of ECC, such as pit and fissure sealing and topical fluoridation, have been vigorously promoted in Guangdong over the past 10 years to reduce the ECC. Moreover, with the marked increase in the development of economy and improvement of people’s living standard in Guangdong during the past 15 years, the factors that impact ECC may have changed [7]. As the economically leading province since 1989, Guangdong has shown a marked increase in development of economy and improvement in people’s living standards, especially during the past ten years [7]. The rise of the per capita GDP (¥24,647 to ¥67,503), the per capita disposable income (¥9730.2 to ¥24,058.8) and the per capita consumption level (¥9799 to ¥26,365) increased significantly from 2005 to 2015 [8]. However, the prevalence of caries of preschool children in Guangdong was among the top ten in 31 provinces of mainland China [9] and was inconsistent with the economic development. Is the caries status of preschool children in Guangdong closed to the goal of a 50% caries-free rate among 5-year-old children? It’s unknown, and it’s unclear how effective the preventive measures are. Another provincial survey of ECC in Guangdong is needed to determine the public health importance and the effect of the preventive measures.

The aim of this study was to examine the prevalence and severity of ECC among preschool children from Guangdong Province, Southern China. In addition, to assess the association of ECC with reported oral health-related behaviors.

## Participants, materials and methods

### Selection of participants and sampling design

This cross-sectional survey was conducted during December 2015 and April 2016. Preschool children residing in Guangdong were enrolled in this study. The participants were divided into three groups according to their ages (3-, 4-, and 5-year-olds). The ratio of males to females in each group was 1:1. Half of the participants were derived from urban areas, while the other from rural areas. The sample size was calculated by the N = deff μ ${}_{\alpha /2}^2$ p(1-p)/δ^2^ formula. Considering the stratification of urban-rural and male-female, the sampling design efficiency ‘deff’ was set to 4.5, and the test level ‘α’ was set to 0.05, so μ _*α*/2_ was equal to 1.96 (here, 2.0 was used), the expected prevalence ‘p’ was set to 0.678 [4], and the allowable error ‘δ’ was set to 0.10p. The theoretical sample size in this designated study was at least 855 after original calculation. The actual sample size was adjusted to 864 for ease of further grouping and implementation. To recruit the participants, an equal-sized, stratified, multistage, random sampling approach (probability proportional to size, PPS) was used. First of all, four districts together with four counties from Guangdong Province were randomly chosen using a PPS method. Second, three kindergartens were selected from either one district or one county with the PPS method. Finally, 36 children in each age group from the chosen kindergartens were recruited with a quota sampling approach. Recruitment was stopped after reaching the study size.

The inclusion criteria of the present study was applicable for both children who participated in the study and its legal guardians. Children must be in a designated range of age, have a willingness to cooperate with the investigators, and the ability to maintain good compliance. Legal guardian’s inclusion criteria included the ability to understand the study as well as the questionnaire and willingness to sign the relevant informed consent letter. The exclusion criteria in our survey were: legal guardian’s failure in understanding this survey; the presence of oral disease requiring medication or even emergency treatment in the children, or the presence of a major systematic disease, which has an impact on the data integrity and/or the safety of the participant.

### Ethics approval and consent to participate

This study was approved by the Stomatological Ethics Committee of the Chinese Stomatological Association on July 9, 2014 (Approval No.: 2014–003). Written informed consents were obtained from the participants’ legal guardians before the survey.

### Clinical examination

The presence of ECC was determined using the dmft index of the WHO 2013 criteria [10]. Three experienced dentists collected the data at kindergartens. The dentists were trained and calibrated using clinical intraoral photographs before the formal examination. And the training of the investigators were conducted with a group of samples (15 volunteers) under the guidance of a standard examiner of the Fourth National Oral Health Survey. The kappa scores of the three examiners to the standard examiner were 0.80, 0.83, and 0.88 when the training finished.

All of the participants were subjected to a general tooth examination using a CPI probe and a plane mirror with an LED light. ECC was diagnosed at cavitation level, which was confirmed by the ball-end CPI probe. The teeth were cleaned with a cotton swab before the examination. The inter-examiner reproducibility was assessed by re-examination of approximately 5% of the participants.

### Questionnaire

Additionally, at a face-to-face and one-on-one interview by trained interviewers, each of the participants’ parents or grandparents completed a questionnaire, which included data regarding caries-related oral hygiene behaviors, dietary behaviors, socioeconomic factors. The questionnaire was from the Fourth National Oral Health Survey, which was designed based on previous studies with purpose of identifying factors related to ECC and was proved to be with appropriate reliability and validity before the study. An additional file shows this in more detail [see Supplementary file 1]. Before the interview, the kindergarten teachers had carried out publicity, education and liaison work; during the interview, we started from caring for the health of children, and patiently explained the work to dispel the doubts of the participants’ parents/grandparents, to handling potential bias.

### Statistical analysis

SAS 9.4 software (SAS Institute, Inc., Carey, NC, USA) was applied. Differences between different groups were compared by *t-*test, chi^2^ test, and One-way ANOVA. Estimates were significantly different if 95% CIs do not overlap. Multivariate logistic regression was used to assessed the percentages of participants with ECC relative to caries-related factors whose *P*-values were less than 0.2 in the bivariate analyses. Odds ratios (ORs) with 95% confidence intervals (CIs) were thus reported. Estimates were significantly different from the reference if its 95%CIs do not include 1. Furthermore, a covariance analysis model was constructed to explore the relationship between dmft and the relevant factors. Estimates were significantly different from the reference if its 95%CIs do not include 0.

The analyses were weighted by age, gender and residence in order to avoid possible non-response bias. Each participant had a personal dmft score (0–20). Prevalence was evaluated by the percentage of participants with a personal dmft score (> 0). The average of the total dmft score of all participants was described as the mean dmft. The filled rate was the ratio of the filled teeth to the total dmft (proportion of ft. in dmft).

## Results

The study sample comprised 2592 participants. The prevalence, mean dmft and filled rate of ECC among 3- to 5-year-old children in Guangdong Province are shown in Table 1. The prevalence and mean dmft in rural areas were higher than those in urban areas, and there was an increasing trend with age. The filled rate in rural areas was lower than that in urban areas. The results did not indicate obvious gender differences.

**Table 1:**
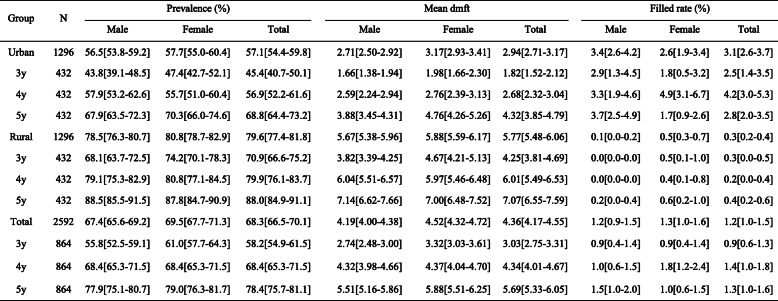
The status of ECC of different ages, genders, residences (weighted, 95%CI)

During the examination, interexaminer kappa values of 0.98, 0.96, and 0.97 were obtained. The interexaminer reproducibility was completely reliable.

**Fig. 1:**
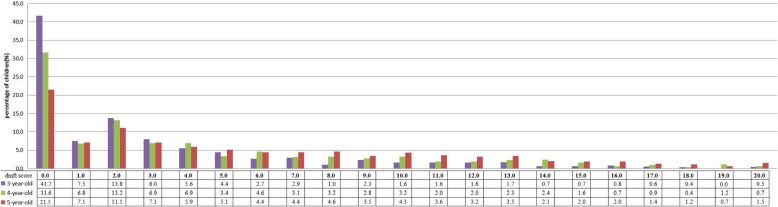
The frequency distribution of dmft. The purple column illustrates the percentage of children (dmft) of 3-year-olds. The green column illustrates the percentage of children (dmft) of 4-year-olds. The brown column illustrates the percentage of children (dmft) of 5-year-olds

The frequency distribution of dmft is shown in Fig. 1. Among the children with ECC, children who had two teeth with ECC were the most common. With the increase in dmft scores, the number of children gradually decreased. A total of 84.8, 79.8 and 72.3% of ECC cases were concentrated in one-third of 3-, 4- and 5-year-old children, and the mean dmft values for these children (significant caries index, SiC) were 7.71 (95% CI: 7.22–8.20), 10.40 (95% CI: 9.94–10.86) and 12.35 (95% CI: 11.96–12.74), respectively.

**Fig. 2:**
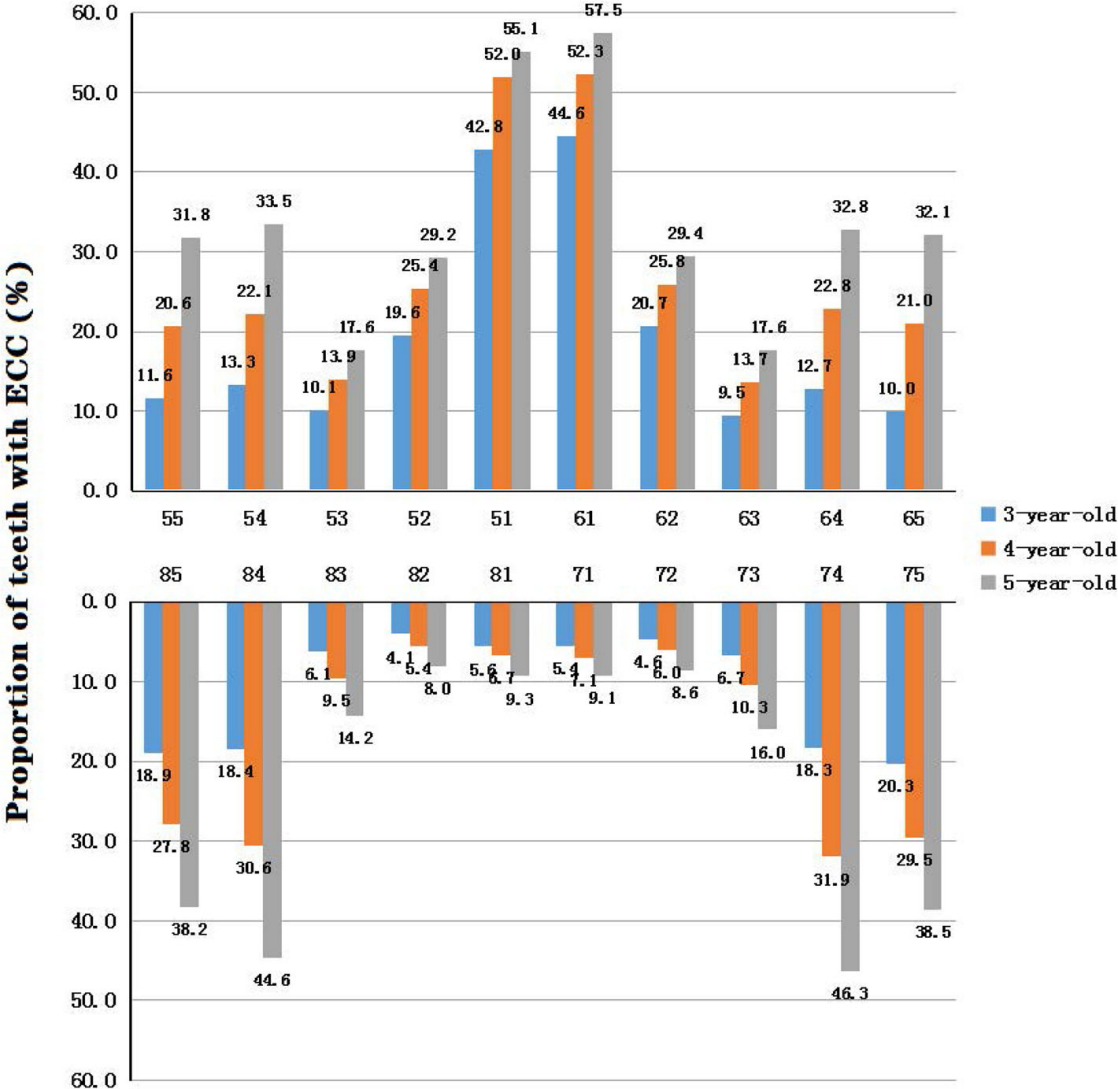
The distribution of teeth with ECC by tooth position. The blue column illustrates the proportion of teeth with ECC among 3-year-olds. The orange column illustrates the proportion of teeth with ECC among 4-year-olds. The gray column illustrates the proportion of teeth with ECC among 5-year-olds

The distribution of the tooth position of ECC is shown in Fig. 2. The upper central incisors had the highest prevalence, followed by the mandibular molars and then the maxillary molars, while the mandibular incisors had the lowest prevalence.

The results of the multivariate analysis are shown in Table 2. Associated factors for both prevalence and mean dmft were older age, rural areas, consumption of sweets before sleep, dental visit history, low household income, and low parental education level. Initiating toothbrushing after 3 years of age and being exclusively/ predominantly breastfed indicated only the prevalence, whereas being female and being frequently fed with sweetened milk/powdered milk indicated only the mean dmft.

**Table 2:**
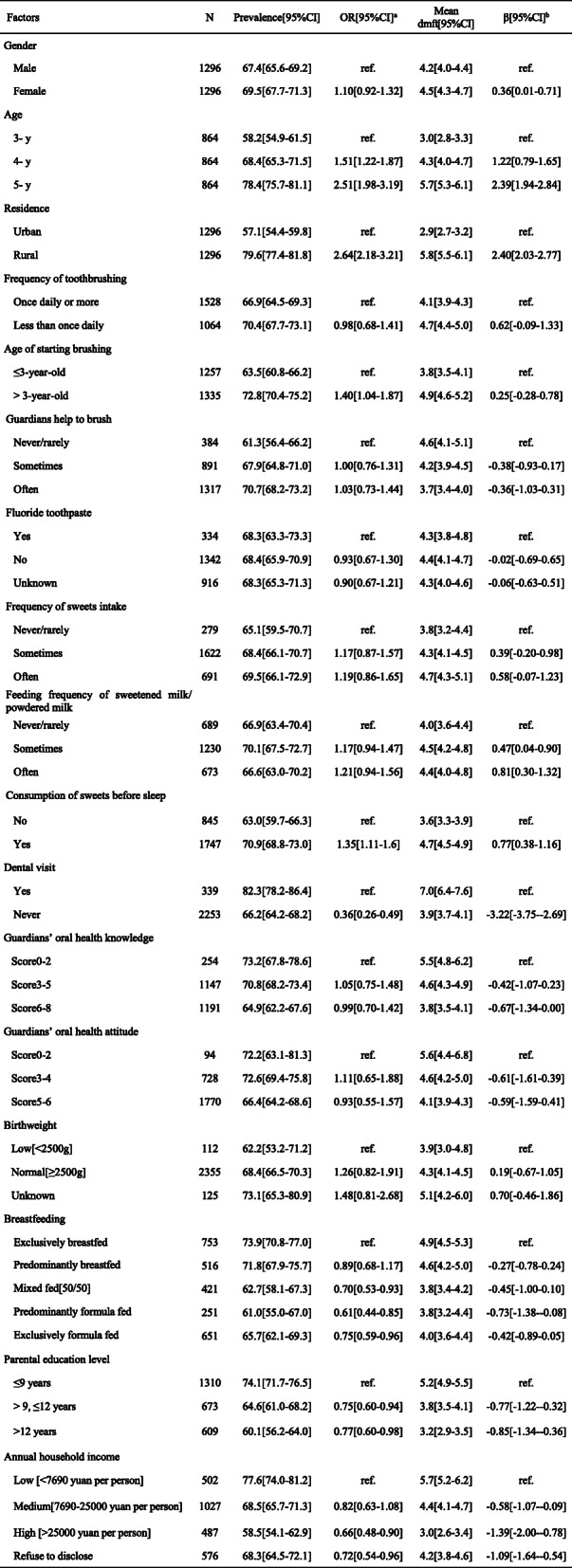
The results of the multivariate analysis: the associated factors of ECC among 3-to 5-year-old children

## Discussion

This survey reported high prevalence and severity of ECC among preschool children in Guangdong, Southern China, similar to the finding for East China [11] and higher than that for Southwest China [12], higher than the national average [13], and higher than that 10 years ago [4]. A multitude of factors are associated with the prevalence and severity. The findings of this survey inform target factors for collective actions to address this critical oral health problem.

The most obvious limitation of this survey is the retrospective rather than prospective design, which might brought potential recall bias. Hence, we can’t confirm the causal relationship between the associated factors and the results. An additional limitation is that the dmft index is less accurate than the dmfs index, as a tooth has multiple tooth surfaces and ECC typically involves multiple tooth surfaces rather than one. Finally, enamel defects, oral bacteria and fluoride concentrations of drinking water that contribute to ECC were not evaluated. A systematic review showed that the presence of enamel defects and high levels of *mutans streptococci* were the strongest risk factors associated with ECC [14]. Prospective studies and studies including microbiological detection, enamel defect examination, and determination of the fluorine concentration in drinking water are indicated.

A report showed that the oral health status of preschool children has significantly improved over the past 10 years in China [15]. However, compared with the provincial survey of Guangdong in 2005, the current results showed remarkable increases in the prevalence (67.9 to 78.4%) and mean dmft (4.13 to 5.69) of ECC among 5-year-old children in both urban and rural areas, especially in rural areas. This trend actually shows ECC rates becoming further away from the goal of a caries-free rate of 50% among 5-year-old children for the year 2020 by the WHO [6]. Higher sugar intake [16] but still inadequate oral healthcare [7], possibly leading to the increasing trend observed [17]. More attention should be given in Guangdong to children’s dental health for deciduous teeth.

The frequency distribution of dmft showed that two teeth with ECC were the most common, and the position distribution showed that the upper central incisors were the prevailing teeth with ECC. The same finding was shown in the national survey [18]. ECC usually affects the smooth surface, followed by the pit and fissure, which is different from the caries of permanent dentition. Therefore, preventive measures against caries with smooth surfaces should be taken. If ECC of the upper central incisors were prevented, the prevalence might be reduced by 10%.

Risk assessment is an important part of patient-centered, evidenced-based decision making in modern medical services [19]. A systematic review showed that ECC is related to demographic factors, oral hygiene behaviors, dietary behaviors, and socioeconomic factors [20]. The results of the multivariable model showed that associated factors for ECC matched the review.

Dental caries is an irreversible process. As deciduous teeth continue to function and be challenged before replacement, the continuous and cumulative effects cause ECC to develop with time. This might explain why increased age was related to a higher risk and severity of ECC. Additionally, children who initiate toothbrushing at an older age have a higher risk of developing ECC. Therefore, ECC prevention should occur from an early age.

No obvious gender differences in the prevalence of ECC were found for. No evidence showed that the composition of the saliva differs between boys and girls or that there are any differences in tooth structure between the genders [21]. However, the mean dmft was higher in female children. Further exploration is indicated.

Generally, caries are recognized as more severe among people with low socioeconomic status [22, 23]. In this study, the prevalence and mean dmft of ECC in urban areas were lower than those in rural areas, while the opposite was true for the filled rate. Children who had a low parental education level and low household income had higher prevalence and severity of ECC. Rural oral medical service is usually characterized as having insufficient infrastructure, socioeconomic difficulties, artificial barriers such as distance and inconvenience of transport, and a high patients to oral health professionals ratio [15, 24]. Parental factors such as education and income are significant risk factors in the developing of ECC [25]. Therefore, children from rural areas should be given more attention in order to reduce ECC.

A systematic review showed that ECC is related to frequent sweets consumption [26]. In particular, numerous studies have proven that sweets play a key role in the occurrence and development of ECC [27–29]. A diet rich in exogenous sugars is believed to be cariogenic to children [30]. In the present survey, the participants who consumed sweets before sleep were 1.35 times more likely to have ECC and had 0.77 higher dmft than those without this habit. Moreover, children who were sometimes/often fed with sweetened milk/powdered milk had higher dmft than those who were never/rarely fed with that kind of milk. This result suggests that both the timing and frequency of the consumption of sweets influenced ECC.

What interested us was that the children who were exclusively/predominantly breastfed during the first half year of life had a higher risk of ECC. This finding agrees with the results of earlier studies reporting a significant relation between the duration of breastfeeding and ECC in young Chinese children [31, 32], similar to its neighboring province of Zhejiang [11]. Part of the reason might be that breast milk is more likely to cause caries than other types of milk [33]. Additionally, extended breastfeeding contributes to the colonization of *Streptococcus mutans* [34], which synergistically induces ECC. Breastfeeding is recommended by the WHO [35]; however, considering the relationship between breastfeeding and ECC, dental experts recommended weaning from the breast shortly after the child’s first birthday [36].

It is recommended that the first dental visit should be performed by 12 months to assess the risk of ECC and provide early intervention when needed [37]. Dental visit history is usually a protective factor for ECC in developed areas [38, 39]. However, in this survey, children who had dental visit histories had higher prevalence and dmft scores of ECC than those without dental visits. This difference may be due to the different patterns of visiting dentists. Children in developed areas often visit dentists for preventive examinations and take preventive measures. However, in China, dental treatment has been sought since these children had already experienced oral health problems, which is therapeutic rather than preventive. Visiting dentists only when a problem was perceived was a risk factor for ECC [40]. This finding agrees with the results of some other Chinese studies [32, 41, 42].

There were no significant relations between toothbrushing habits and ECC in this study. However, children who start brushing their teeth at an older age are at higher risk of developing ECC. Moreover, lack of toothbrushing was a risk factor for ECC [43], and irregular brushing at 18 months of age was a highly significant predictor of developing ECC [44]. Brushing less than twice a day or difficulties brushing teeth during the first year of preschool were significant determinants of ECC at the age of 5 years [45]. Therefore, professionals should give parents special attention and assist in improving and optimizing their toothbrushing behavior during children’s preschool years.

The strengths of this survey include a representative sample which was ensured by an equal-sized, stratified, multistage random sampling approach, the use of a reliable index, and a statistical analysis with a weighted process which made the results be close to reality and permit us to generalize the findings to the population.

## Conclusion

In conclusion, preschool children in Guangdong Province, especially children from rural areas, experienced a significant amount of ECC. Associated factors for ECC included demographics, oral health measures, dietary factors, and socioeconomic factors. More attention should be given to prevention of ECC from early life. The construction of social support for oral health should be strengthened. Oral health education and promotion, especially of rural areas, should be intensified to reduce the inequality between urban and rural areas.

## Supplementary information

**Additional file 1.** The Questionnaire of the oral health survey, the version for children’s guardians (an English language version, along with the original version).

## Data Availability

The data sets used and/or analyzed during the current study are available from the corresponding author on reasonable request.

## Abbreviations

CI(s): Confidence Interval(s)
CPI: Community Periodontal Index
dmfs: decayed, missing, filled deciduous tooth surface
dmft: decayed, missing, filled deciduous teeth
ECC: Early Childhood Caries
GDP: Gross Domestic Product
OR(s): Odds Ratio(s)
PPS: Probability Proportional to Size
SiC: Significant Caries Index
