# Supplementary material for: The status and associated factors of early childhood caries among 3- to 5-year-old children in Guangdong, Southern China: a provincial cross-sectional survey
Source: BMC Oral Health. 2020 Sep 25;20:265. doi: 10.1186/s12903-020-01253-w (PMC7517683; doi:10.1186/s12903-020-01253-w)
Supplement: Supplementary file 1 — Additional file 1. The Questionnaire of the oral health survey, the version for children’s guardians (an English language version, along with the original version). [file 12903_2020_1253_MOESM1_ESM.pdf]

Oral Health Questionnaire for Guardians of Children

Respondent ID：□□□□□□□□□□□ **Name of the child**：________

year month day

Survey Date：201□--□□--□□ Investigator NO.：□

Note: Only the child's parents and grandparents can complete this questionnaire!

Requirements: Please mark “ √ ” in the “□” in front of the corresponding option of the selected question.

1.What is your relationship with the child? （**Choose only one answer**）

1)□ Father 2)□ Mother 3)□ Grandfather/Maternal Grandfather

4)□ Grandmother/Maternal Grandmother

2.The weight of your child at birth is__Semi kg。**(Please keep one decimal place, fill in "N" if you don't know or refuse to answer)**

3.How was your child fed within six months after birth? **(Choose only one answer)**

1)□ Exclusively Breastfed 2)□ Predominantly Breastfed

3)□ Exclusively Formula Fed 4)□ Predominantly Formula Fed

5)□ Mixed Fed[50/50]

4.How often does your child eat or drink any of the following foods or beverages? **(choose one answer for each question)**

	6<br>Twice and above a day	5<br>Once daily	4<br>Twice to six times a week	3<br>Once a week	2<br>Once to three times a month	1<br>Rarely/ Never
1）Sweet desserts(biscuits, cakes, bread) and candy(chocolate, sugary gum)	□	□	□	□	□	□
2）Sweet drinks (sweet water, cola and other carbonated beverages, orange juice, apple juice and other juices, lemonade and other non-fresh juices)	□	□	□	□	□	□
3) milk, yogurt, milk powder, tea, soy milk, coffee containing sugar	□	□	□	□	□	□

5.Does your child eat dessert or drink sweet drinks before going to bed at night? **(Choose only one answer)**

1) ☐ Often 2) ☐ Occasionally 3) ☐ Never

6. Does your child brush their teeth? **(Choose only one answer)**

1) ☐Yes 2) ☐Occasionally or Never(choose 2 will not answer questions 7 to 11)

7. At what age does your child start brushing teeth? **(Choose only one answer)**

1) ☐ Half a year old 2) ☐ 1 year old 3) ☐ 2 years old

4) ☐ 3 years old 5) ☐ 4 years old 6) ☐ 5 years old

7) ☐ Don't remember

8. How often does your child brush his/her teeth every day? **(Choose only one answer)**

1) ☐ Twice and Above 2) ☐ Once 3) ☐ Not Every Day

9. Do you help your child brush his/her teeth? **(Choose only one answer)**

1) ☐ Every Day 2) ☐ Every Week 3) ☐ Sometimes

4) ☐ Occasionally 5) ☐ Never

10. Does your child use toothpaste when brushing his/her teeth? **(Choose only one answer)**

1) ☐ Yes 2) ☐ No

3) ☐ Don't know (choose 2 or 3 will not answer question 11)

11. Does your child use toothpaste that contains fluoride when brushing his/her teeth? **(Choose only one answer)**

1) ☐ Yes 2) ☐ No 3) ☐ Don't know

12. During the past 12 months, how often did your child have toothache or feel discomfort due to his/her teeth? **(Choose only one answer)**

1) ☐ Never 2) ☐ Sometimes 3) ☐ Often 4) ☐ Not Clear

13. Did your child ever go to the dentist? **(Choose only one answer)**

1) ☐ Yes 2) ☐ Never (choose 2 will not answer questions 14 to 17)

14. How long is it since your child last saw a dentist? **(Choose only one answer)**

1) ☐ Within 6 months 2) ☐ 6-12 months 3) ☐ More than 1 year (choose 1 or 2 will not answer question 18, chooses 3 will not answer questions 15 to 17)

15. What was the reason of your child last visit to the dentist? **(Choose only one answer)**

1) ☐ Consultation/Routine check-up 2) ☐ Prevention

3) ☐ Treatment 4) ☐ Don't know

16. How much did your child cost due to the dental visit in the past year?￥____yuan **(Please fill in an integer, if you don't know or refuse to answer, fill in "N")**

17. Among the fees for dental care mentioned above, the proportion that you personally need to pay was ____%. **(Please fill in an integer, if you don't know or refuse to answer, fill in "N")**

18. Why did your child not go to see a dentist during the past 12 months? **(Optional multiple answers)**

1) ☐ The child's teeth were OK 2) ☐ The child's teeth were not serious

3) ☐ The deciduous teeth need to be replaced, so treatment wasn't needed

4) ☐ Financial difficulties, could not afford 5) ☐ Not convenient to see a dentist

6) ☐ Too busy, No time 7) ☐ The child was afraid of pain of treatment

8) ☐ There was no dentist nearby 9) ☐ Fear of infectious diseases

10) ☐ It was difficult to find a trusted dentist 11) ☐ It was too difficult to register

12) ☐ Dental treatment in kindergarten 13) ☐ Other reasons

19. How would you describe the general health of your child? **(Choose only one answer)Average**

1) ☐ **Very Good** 2) ☐ **Good** 3) ☐ **Average** 4) ☐ **Poor** 5) ☐ **Very Poor**

20. How would you describe the dental health and oral health of your child? **(Choose only one answer)**

1) ☐ **Very Good** 2) ☐ **Good** 3) ☐ **Average** 4) ☐ **Poor** 5) ☐ **Very Poor**

21. What is your opinion on the following statement? **(Choose one answer for each question)**

	1<br>Correct	2<br>Incorrect	8<br>Don't know
1)Bleeding gums when brushing is normal	☐	☐	☐
2)Bacteria can cause inflammation of gums	☐	☐	☐

3)Tooth-brushing is useless to prevent bleeding gums	☐	☐	☐
4) Bacteria can cause dental caries	☐	☐	☐
5) Eating sugar may cause dental caries	☐	☐	☐
6) The broken deciduous teeth need to be replaced, so treatment isn’t needed	☐	☐	☐
7) Pit and fissure sealing can prevent dental caries	☐	☐	☐
8) Fluoride is useless for tooth protection	☐	☐	☐

23. What level of education have you completed? (Choose only one answer)

1) ☐ No formal schooling　　2) ☐ No more than primary school

3) ☐ No more than junior school　　4) ☐ No more than High school

5) ☐ No more than secondary school　　6) ☐ No more than College

7) ☐ No more than university　　8) ☐ Postgraduate degree

24. How many people live together in your family? _______Person(s) **(please fill in an integer, fill in "N" if you don't know or refuse to answer)**

25. What was the total household income in the past 12 months?

￥_________10,000 yuan/year **(please fill in an integer, fill in "N" if you don't know or refuse to answer)**

Thank you very much for your cooperation!

调查问卷

2015年第四次全国口腔健康调查问卷（儿童家长）

被调查者 ID 号：□□□□□□□□□□□　　　**被检查儿童姓名：______**

调查日期：201□年□□月□□日　　　**调查员编号：□**

注意：只有孩子的父母和祖父母/外祖父母才能完成本问卷！ 要求：请在选 择题相应选项前面的"□"内划"√"。

1. 您是孩子的？**（只选一个答案）**

   1) □父亲　2) □母亲　3) □祖父/外祖父　4) □祖母/外祖母

2. 您孩子出生时的体重是__斤。**(请保留一位小数，不知道或拒绝回答的填写"N"）**

3. 您孩子出生后六个月内喂养的方式？**（只选一个答案）**

   1) □完全母乳喂养　2) □母乳喂养为主
   3) □完全人工喂养　4) □人工喂养为主
   5) □母乳喂养和人工喂养各半

4. 您孩子平时进食以下食品或饮料的频率如何?**（每小题选一个答案）**

	6 每天 ≥2次	5 每天 1次	4 每周 2-6次	3 每周 1次	2 每月 1-3次	1 很少/ 从不
1）甜点心（饼干、蛋糕、面包）及糖果（巧克力、含糖口香糖）	□	□	□	□	□	□
2）甜饮料（糖水、可乐等碳酸饮料，橙汁、苹果汁等果汁、柠檬水等非鲜榨果汁）	□	□	□	□	□	□
3）加糖的牛奶、酸奶、奶粉、茶、豆浆、咖啡	□	□	□	□	□	□

5. 您孩子在晚上睡前吃甜点或喝甜饮料吗？**（只选一个答案）**

   1) □经常　2) □偶尔　3) □从不

6. 您孩子刷牙吗？**（只选一个答案）**

   1）□刷牙　2）□偶尔刷或从不刷**（选 2 项者不回答第 7 至 11 题）**

7. 您孩子从几岁开始刷牙？**（只选一个答案）**

   1) □半岁　2) □1岁　3) □2岁
   4) □3岁　5) □4岁　6) □5岁　7) □不记得

8. 您孩子每天刷几次牙？（**只选一个答案**）

1) □2次及以上　　2) □1次　　3) □不是每天刷

9. 您帮助孩子刷牙吗？（**只选一个答案**）

1) □每天　　2) □每周　　3) □有时　　4) □偶尔　　5) □从没做过

10. 您孩子刷牙时用牙膏吗？（**只选一个答案**）

1）□是　　2) □否　　3）□不知道（**选 2 或 3 项者不回答第 11 题**）

11. 您孩子刷牙时用含氟牙膏吗？（**只选一个答案**）

1) □是　　2) □否　　3) □不知道

12. 在过去的12个月内，您孩子是否有过牙痛或不适？（**只选一个答案**）

1) □从来没有　　2) □有时候有　　3) □经常有　　4) □不清楚

13. 您孩子去医院看过牙吗？（**只选一个答案**）

1）□看过　　2) □从来没看过（**选 2 项者不回答第 14 至 17 题**）

14. 您孩子最近一次去医院看牙距离现在多长时间？（**只选一个答案**）

1）□ 6个月以内　　2）□ 6个月至12个月（**选1或2项者不回答第18题**）

3）□ 12个月以上（**选 3 项者不回答第 15 至 17 题**）

15. 您孩子最近一次去医院看牙的主要原因是什么？（**只选一个答案**）

1) □咨询检查　　2) □预防　　3) □治疗　　4) □不知道

16. 在过去的一年内您孩子去医院看牙的总费用是___元？（**请填一个 整数，不知道或拒绝回答的填写"N"**）

17. 在上述看牙费用中，您个人需要支付的比例是____%。（**请填一个整数, 不知道或拒绝回答的填写"N"**）

18. 您孩子在过去12个月里没有去医院看牙的原因是？（**可选多个答案**）

1) □孩子的牙没问题　　2) □孩子的牙坏得不严重

3) □乳牙要替换，不需要看　　4) □因为经济困难，看不起牙

5) □看牙不方便　　6) □太忙、没时间

7) □孩子害怕看牙疼痛　　8) □附近没有牙医

9) □害怕传染病　　10) □很难找到信得过的牙医

11) □挂号太难　　12) □在幼儿园看牙　　13) □其它原因

19. 您对孩子的全身健康状况评价如何？（**只选一个答案**）

1）□很好　　2）□较好　　3）□一般　　4）□较差　　5）□很差

20. 您对孩子的牙齿和口腔状况评价如何？（**只选一个答案**）

1）□很好　2）□较好　3）□一般　4）□较差　5）□很差

21. 您对以下说法的看法如何？**(每小题选一个答案)**

	1<br>同意	2<br>不同意	8<br>无所谓	9<br>不知道
1）口腔健康对自己的生活很重要	□	□	□	□
2）定期口腔检查是十分必要的	□	□	□	□
3）牙齿的好坏是天生的，与自己的保护关系不大	□	□	□	□
4）预防牙病首先靠自己	□	□	□	□
5）保护孩子六龄牙很重要	□	□	□	□
6）母亲牙齿不好会影响孩子的牙齿	□	□	□	□

22. 您认为下面的说法是否正确？**(每小题选一个答案)**

	1<br>正确	2<br>不正确	8<br>不知道
1）刷牙时牙龈出血是正常的	□	□	□
2）细菌可以引起牙龈发炎	□	□	□
3）刷牙对预防牙龈出血没有用	□	□	□
4）细菌可以引起龋齿	□	□	□
5）吃糖可以导致龋齿	□	□	□
6）乳牙坏了不用治疗	□	□	□
7）窝沟封闭能预防儿童龋齿	□	□	□
8）氟化物对保护牙齿没有用	□	□	□

23. 您获得的最高学历是什么？（**只选一个答案**）

1)□没有上过学　2)□小学　3)□初中　4)□高中
5)□中专　6)□大专　7)□本科　8)□硕士及以上

24. 您家里共同生活的有几口人？__人（**请填一个整数，不知道或拒绝回答的填写“N”**）

25. 您家共同生活的人在过去的 12 个月内的总收入是多少？___万元/ 年(请填一个整数，不知道或拒绝回答的填写“N”）

十分感谢您的合作！
